# Expression and Characterization of Relaxin Family Peptide Receptor 1 Variants

**DOI:** 10.3389/fphar.2021.826112

**Published:** 2022-01-28

**Authors:** David Speck, Gunnar Kleinau, Mark Meininghaus, Antje Erbe, Alexandra Einfeldt, Michal Szczepek, Patrick Scheerer, Vera Pütter

**Affiliations:** ^1^ Charité – Universitätsmedizin Berlin, Corporate Member of Freie Universität Berlin and Humboldt-Universität zu Berlin, Institute of Medical Physics and Biophysics, Group Protein X-ray Crystallography & Signal Transduction, Berlin, Germany; ^2^ Bayer AG, Research and Development, Pharmaceuticals, Wuppertal, Germany; ^3^ Bayer AG, Research and Development, Pharmaceuticals, Berlin, Germany; ^4^ NUVISAN ICB GmbH, Berlin, Germany; ^5^ DZHK (German Centre for Cardiovascular Research), partner site Berlin, Berlin, Germany

**Keywords:** G-protein coupled receptors (GPCR), leucine-rich repeat containing receptor 7 (LGR7), relaxin family peptide receptor 1 (RXFP1), fluorescence-detection size-exclusion chromatography (FSEC), protein engineering, surface plasmon resonance spectroscopy (SPR)

## Abstract

G-protein coupled receptors (GPCR) transduce extracellular stimuli into the cell interior and are thus centrally involved in almost all physiological-neuronal processes. This essential function and association with many diseases or pathological conditions explain why GPCRs are one of the priority targets in medical and pharmacological research, including structure determination. Despite enormous experimental efforts over the last decade, both the expression and purification of these membrane proteins remain elusive. This is attributable to specificities of each GPCR subtype and the finding of necessary experimental *in vitro* conditions, such as expression in heterologous cell systems or with accessory proteins. One of these specific GPCRs is the leucine-rich repeat domain (LRRD) containing GPCR 7 (LGR7), also termed relaxin family peptide receptor 1 (RXFP1). This receptor is characterized by a large extracellular region of around 400 amino acids constituted by several domains, a rare feature among rhodopsin-like (class A) GPCRs. In the present study, we describe the expression and purification of RXFP1, including the design of various constructs suitable for functional/biophysical studies and structure determination. Based on available sequence information, homology models, and modern biochemical and genetic tools, several receptor variations with different purification tags and fusion proteins were prepared and expressed in *Sf9* cells (small-scale), followed by an analytic fluorescence-detection size-exclusion chromatography (F-SEC) to evaluate the constructs. The most promising candidates were expressed and purified on a large-scale, accompanied by ligand binding studies using surface plasmon resonance spectroscopy (SPR) and by determination of signaling capacities. The results may support extended studies on RXFP1 receptor constructs serving as targets for small molecule ligand screening or structural elucidation by protein X-ray crystallography or cryo-electron microscopy.

## Introduction

In 1926, Frederick Hisaw set the starting point for relaxin research when he transferred blood plasma from a pregnant to a virgin guinea pig, thereby triggering pubic ligament relaxation in the recipient animal (Bylander et al., 1987). The relaxin sequence was determined in 1984 (Fields and Larkin, 1981; Yamamoto et al., 1981; Hudson et al., 1984) after successful isolation from the decidua and placenta. Following several years, the corresponding relaxin binding partner was identified (Hsu et al., 2000; Hsu et al., 2002). Based on sequence similarities with previously discovered leucine-rich repeat containing receptors (LGRs), this relaxin receptor was originally termed LGR7 but later renamed RXFP1 (Braun et al., 1991; Hsu et al., 1998; Bathgate et al., 2006). RXFP1 was traditionally purported to be involved in pregnancy and parturition processes; however, a sex-independent receptor-transcript distribution has been shown (e.g., kidney, brain, and cardiac cells) (Kohsaka et al., 1998; Hsu et al., 2000; Hsu et al., 2002; Samuel et al., 2004a; Luna et al., 2004), provoking the assumption of a variety of different physiological functions. Knock-out of the *Lgr7* (*Rxfp1*) gene in mice, as observed for the relaxin knock-out, resulted in prolonged birth duration and increased infant mortality (Kamat et al., 2004; Krajnc-Franken et al., 2004). Receptor or ligand knock-out also led to an age-dependent increase in tissue fibrosis, whereby relaxin was studied extensively as an anti-fibrotic therapeutic (Samuel et al., 2003; Samuel et al., 2004a; Samuel et al., 2004b; McBride et al., 2017; Samuel et al., 2017).

RXFP1 activates intracellularly at least three different Gα variants, namely Gα_s_, Gα_i_, and Gα_o_ (Hsu et al., 2000; Hsu et al., 2002; Halls et al., 2006; Halls et al., 2009a; Halls et al., 2009b), whereby the type of associated G-protein is also dependent on the cell type and receptor expressing tissue. Finally, RXFP1 and identified splicing variants are recognized as important key players in physiology and are also related to various disease states. Generally, RXFP1 or receptor variants are involved in cancer development, as well as skin, kidney, cardiac, liver, and lung fibrosis (reviewed in Chen et al. (2020).

RXFP1 is classified as a class A rhodopsin-like GPCR (Petrie et al., 2015). The superfamily of GPCR in humans comprises more than 800 members. They transduce a huge diversity of extracellular stimuli into the cell interior for cell-signaling regulation (Limbird, 2004). GPCRs are related to more than 100 human diseases, including cancer and endocrine pathologies (O'Hayre et al., 2013; Schöneberg et al., 2004; Vassart and Costagliola, 2011). This fact, combined with their omnipresence in nearly all physiological processes, reasons why these receptors are a strong focus of academic research or pharmacological investigations, including structure determination (Heyder et al., 2021) and drug development (Garland, 2013; Errey and Fiez-Vandal, 2020).

**FIGURE 1:**
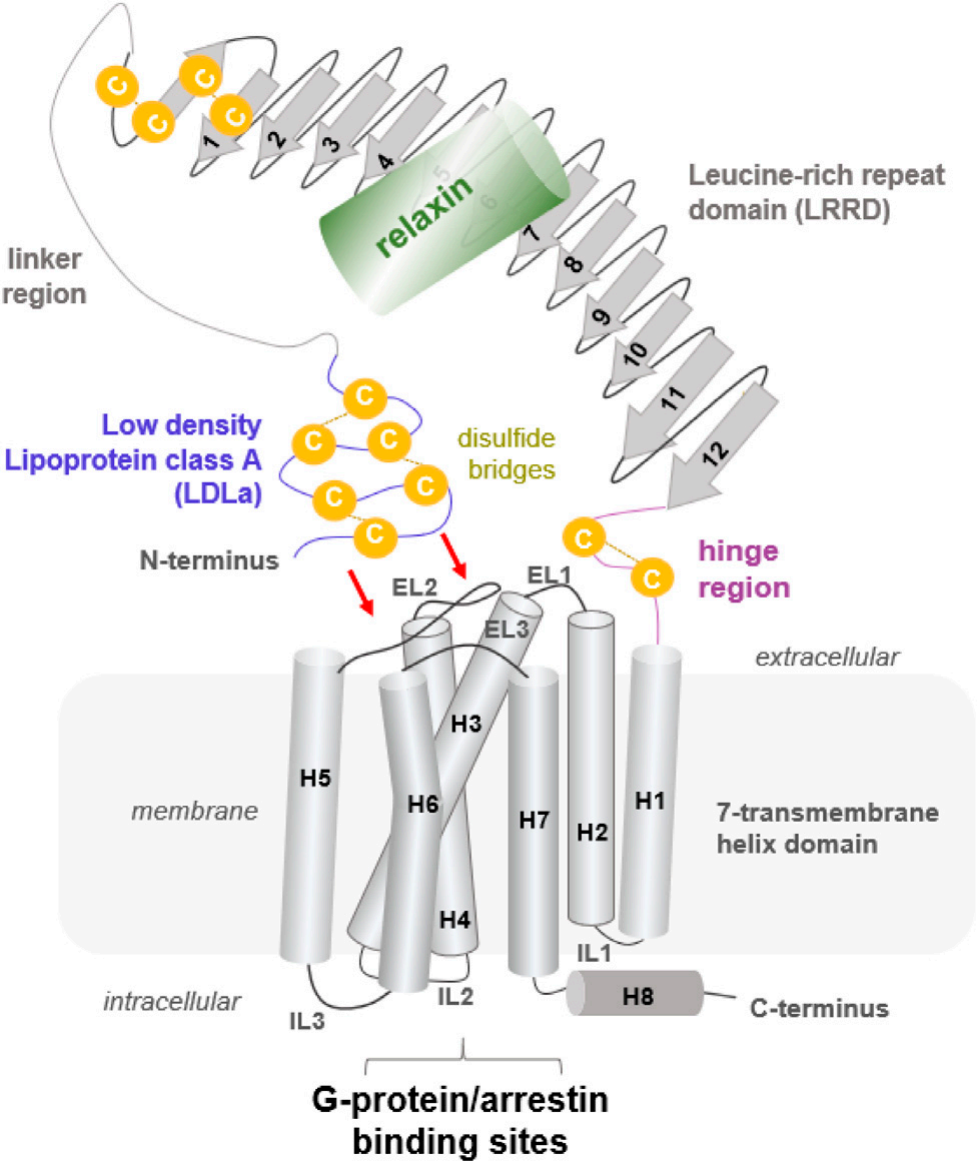
Putative structural arrangement of the different putative RXFP1 components. The RXFP1 receptor shows typical GPCR features as the seven transmembrane helices and exposes specific properties significant for LGRs in the class A GPCRs, such as a large extracellular part constituted by several interrelated domains. Their exact spatial arrangement to one another remains unknown, but it is assumed that the N-terminal LDLa domain interacts directly with extracellular loops (indicated by red arrows). The endogenous ligand relaxin, a peptide hormone, binds to the LRRD and simultaneously to the linker region.

RXFP1 and RXFP2 form the LGR group C (Wilkinson et al., 2005). The LGR group A includes the homologous lutropin receptor (LHCGR) (Troppmann et al., 2013), the thyrotropin receptor (TSHR) (Kleinau et al., 2013; Kleinau et al., 2017), and the follitropin receptor (FSHR) (Ulloa-Aguirre and Zariñán, 2016) (Supplementary Figure S1). LGR4−6 constitute LGR group B (Park et al., 2005). LGRs are more complex in their extracellular architecture compared to other class A GPCRs. RXFP1 exposes a large extracellular part (∼400 amino acids) that is constituted by the Leucine-rich repeat domain (LRRD) and the low-density lipoprotein class A (LDLa) domain (Hsu et al., 2000), which are connected via a linker region (Supplementary Figure S1, Figure 1). Such module-like extracellular architecture is found mainly in other GPCR classes as the class B (secretin and adhesion) GPCRs (Nordström et al., 2009). Nonetheless, missing structural information for group C LGRs hampers a more complete functional understanding of this receptor. Moreover, only a few peptidic ligands were developed, with most relaxin derivatives (Sudo et al., 2003; Halls et al., 2005; Hossain et al., 2010; Hossain et al., 2011; Chan et al., 2012; Hossain et al., 2016), and only first non-peptidic ligands have been identified, such as the small molecule agonist ML290 (EC_50_ of 94 nM) (Xiao et al., 2010; Xiao et al., 2013; Wilson et al., 2018). Altogether, RXFP1 is a highly interesting GPCR from several aspects; however, considerable detailed information for the comprehensive understanding of this receptor or for modifying its signaling activity remains lacking. Our study aimed to express RXFP1 diverse receptor variants suitable for functional/biophysical studies and structure determination.

## Materials and Methods

### Homology Modelling of the Putative Relaxin Family Peptide Receptor 1 Structure

The human RXFP1 is constituted by 757 amino acids, with half assigned to the N-terminal extracellular part (Kong et al., 2010). A homology model of the RXFP1 was created to predict and visualize putative structural properties and more rationally guide the design of different receptor variants for experimental studies. Moreover, we complexed the RXFP1 model with the ligand relaxin to recapitulate already reported (e.g., (Büllesbach and Schwabe, 2005)) amino acid residues involved in intermolecular interactions.

*Template structures* - The assumed structural architecture of RXFP1 is constituted by several interconnected domains (Kong et al., 2010; Sethi et al., 2016a) (Figure 1). To date, there is very little direct structural information published (Hopkins et al., 2007). For modeling a putative RXFP1 structure, the following templates of evolutionarily related class A GPCRs were used, accompanied by *ab initio* modeling of receptor regions without any template:

(1) For the N-terminal low-density lipoprotein class A (LDLa, amino acids Gly21-Gly63), structural information is available ((Berman et al., 2000), Protein database http://www.rcsb.org ID: 2JM4 (Hopkins et al., 2007)).
(2) The linker region (Asp64-Glu94, numbering with signal peptide) between the LDLa and the LRRD (Figure 1) was added to the LDLa domain without any structural template or structure predictions.
(3) The LRRD was constructed according to the FSHR LRRD structure (PDB ID: 4AY9 (Jiang et al., 2012), RXFP1 sequence Cys95-Ile370).
(4) The extracellular RXFP1 hinge region connecting the putative LRRD and the transmembrane domain (His371-Glu400) was inserted without any template structure.
(5) For modeling the RXFP1 seven transmembrane helix domains (TMD) in an active state conformation (As401-Gln701), the available structural complex of the β2-adrenoreceptor in complex with the Gs protein was utilized (PDB ID: 3SN6 (Rasmussen et al., 2011)).

In addition,

(6) The extracellular loop 2 (EL2) was constructed based on the EL2 of the opsin receptor (PDB ID: 3DQB (Scheerer et al., 2008)) because the amino acid constitution and length of the loop is more similar between opsin and RXFP1 compared to between RXFP1 and the β2-adrenoreceptor (detailed alignment not shown).
(7) The already determined relaxin-3 structure as an evidenced RXFP1 agonist (Sudo et al., 2003) was used as an agonistic peptide ligand (PDB ID: 2FHW, (Rosengren et al., 2006)).
(8) An inactive state RXFP1 model of the TMD structure for comparison with the active state conformation was designed by using the determined inactive state structure of the β2-adrenoreceptor (PDB ID: 2RH1) (Cherezov et al., 2007).

*Template modifications prior to model composition* - Minimal template modifications at the FSHR LRRD for the RXFP1 LRRD structure modeling were manual adjustments in repeat length by deletions or insertions of several amino acids. For modeling the RXFP1 TMD, the β2-adrenoreceptor template was modified by deleting the fused T4-lysozyme, the bound agonistic ligand, and Gs protein. Moreover, the EL1 and EL3 loop lengths were adjusted manually to respective RXFP1 loop lengths. Amino acid residues of the third intracellular loop (IL3) of the RXFP1 were added manually to the template because of the missing IL3 structure in the template. A similar procedure was used to prepare the inactive state conformation of the β2-adrenoreceptor; however, without deletion of the Gs protein.

Further details of modeling methods are provided in the Supplementary Material. Based on this information, we predicted the length and constitution of several designed and tested receptor constructs. These constructs can be constituted by partial receptor variants including specific or single domains, as reported for endogenously expressed splice variants (Chen et al., 2020).

### Cloning of RXFP1 Constructs

Synthetic gene fragments encoding different truncated versions of human RXFP1 protein sequence were integrated into the baculovirus pVL1393 (hereafter abbreviated as pVL) transfer vector or pcDNA6.2 (abbreviated as pcDNA). All constructs (see Figure 4) carried an influenza hemagglutinin secretion tag at the N-terminus followed mostly by a FLAG peptide sequence-tag (FLAG-tag). The C-terminus carried a polyhistidine tag (His-tag) with a subsequent TEV cleavage site, an AVI-tag, and a 1D4-tag. An eGFP-fusion protein-tag was either located at the N-terminus between the FLAG-tag and the receptor (detachable using a PreScission™ protease cleavage site) or at the C-terminus between the TEV cleavage site and the AVI-tag.

### Expression of different RXFP1 Constructs

All constructs were expressed in *Sf9* (*Spodoptera frugiperda*) insect cells (Expression Systems) using the flashBAC baculovirus system (Oxford Expression Technologies) according to the manufacturer’s instructions. *Sf9* cells were cultivated in suspension in ESF921 medium (Expression System).

In 2.5 ml small-scale test expressions, *Sf9* cells were infected at a density of 4.0 x 10^6^ cells/ml with high-titer viral stock at different multiplicities of infection (MOI). Cells were harvested by centrifugation 48 or 72 h after infection and stored at −20°C until purification.

For large-scale recombinant protein production, *Sf9* cells at densities of 4.0 x 10^6^ cells/ml were infected with high-titer viral stock at an MOI of 0.3. Cells were incubated for 48 h at 27°C in a cell wave bag at 22 rpm. Cells were harvested by centrifugation, flash-frozen, and stored at −80°C until use.

### Large-Scale Protein Purification

All purification steps were performed at 4°C or on ice. Biomass corresponding to 2 L of insect cell culture was thawed on ice and resuspended in lysis buffer (50 mM HEPES pH 7.5, 300 mM NaCl, 10% glycerol and 0.1 mM TCEP) supplemented with cOmplete™ protease inhibitor Cocktail (Roche Applied Science). The receptor was solubilized by the addition of 1% (w/v) n-dodectyl-ß-D-maltoside (DDM, Anatrace), 0.1% (w/v) cholesteryl-hemi-succinate (CHS, Sigma), 10 strokes in a glass douncer, followed by 1 h incubation under gentle agitation at 4°C. The insoluble fraction was removed by centrifugation (150,000 rcf, 45 min). Detergent solubilized RXFP1 constructs were captured by gravity flow on 2 ml M2 resin (Anti-Flag M2-agarose, Sigma) pre-equilibrated with solubilization buffer supplemented with 1% DDM (w/v). M2 resin was washed with buffer (50 mM HEPES pH 7.5, 300 mM NaCl, 0.1% DDM, 0.01% CHS, 10% glycerol, and 0.1 mM TCEP) and eluted with the same buffer supplemented with 0.4 mg/ml flag peptide. Immediately after elution, RXFP1 was subsequently concentrated with a 100 kDa Vivaspin Turbo concentrator (Sartorius) to a final volume of 500 µl. The sample was further purified by size-exclusion chromatography using a Superose 6 Increase 10/300 GL column (GE Healthcare) pre-equilibrated with gel filtration running buffer (50 mM HEPES pH7.5, 150 mM NaCl, 0.1% DDM, 0.01% CHS, 10% glycerol, and 0.1 mM TCEP). The monomeric receptor peak was pooled. Aliquots were flash-frozen in liquid nitrogen and stored at −80°C. Binding activity was measured by SPR.

### F-SEC-Analytic Chromatography

Forty microliters of Flag elution fractions were applied on an analytic Superose 6 increase 5/15 GL column (GE Healthcare). From each elution fraction, 5 µl were transferred into a 96-well plate supplemented with a 95 µl gel filtration buffer. The GFP signal of the different fractions was measured in a Tecan M1000 plate reader.

### Thermal Stability Assay Using Nano-Differential Scanning Fluorimetry

The nano Differential Scanning Fluorimetry method (nanoDSF) is based on the intrinsic tryptophan, tyrosine, and phenylalanine fluorescence. This method measures the fluorescence intensity ratio between the fluorescent amino acids in 10 μl capillaries. Once a molecule unfolds, the aromatic amino acid residues sites alter and trigger changes in the fluorescence. The Prometheus NT.48 nanoDSF (NanoTemper Technologies) was used to measure the ratio (350:330 nm) as a function of temperature. In a typical nanoDSF experiment, the intrinsic fluorescence intensity ratio (350:330 nm) is constantly measured and recorded. For analysis of the results, the intrinsic fluorescence intensity ratio (or the first derivative of the ratio) was plotted as a function of temperature, yielding a protein unfolding curve. In the experiment, we used standard glass capillaries from NanoTemper, with a sample volume of a maximum of 10 μl. For each experiment, at least three replicate experiments were performed.

### nanoDSF Data Analysis

nanoDSF data analysis was performed using the PR.ThermControl v2.0.4 software (NanoTemper Technologies). The thermal transition (unfolding) temperature (Tm) was obtained in the post-run data analysis. The Tm values can be used to assess the thermal stability of the analyzed protein.

### Surface Plasmon Resonance Assay

All tested receptor constructs were captured at 20°C via a biotinylated Avi-tag on an SA-chip. Immobilization buffer contained 50 mM HEPES pH 7.5, 150 mM NaCl, 10% glycerol, 0.1% DDM, 0.01% CHS, and 0.1 mM TCEP. The obtained immobilization level was between 3000 and 5000 RU. A Biacore T200 was used for all experiments. Binding assays were performed at 20°C, with running buffer containing 20 mM HEPES pH 7.5, 150 mM NaCl, 0.01% BSA, 0.1% DDM, and 0.01% CHS. Relaxin was injected at a concentration series of 0.41–100 nM. Relaxin H2 (human) from Bachem (#40399910.0200) and R&D Systems (#3596-RN/CF, >97% by SDS-PAGE, activity measured by its ability to induce cAMP accumulation in THP-1 human acute monocytic leukemia cells) was used in SPR and cellular assays, respectively.

### Cell Culture and Transient Transfection of HEK293 Cells

HEK293 cells were cultured in Dulbecco’s modified eagle medium (DMEM) high glucose (Gibco), supplemented with 10% fetal bovine serum, 1.5% HEPES, 1% minimal essential medium non-essential amino acids (MEM NEAA), and 1% sodium pyruvate, in a humidified incubator at 37°C and 5% CO_2_. Twenty-four hours prior to transfection, HEK293 cells were seeded into six-well plates (Cellstar^®^ 6-well Cell culture multiwell plate, Greiner Bio-One) with 5 x 10^5^ cells per well in 5 ml cell culture medium, and incubated at 37°C and 5% CO_2_. Transfection was performed using GenJet (Signagen) according to the manufacturer’s protocol using 2 µg DNA in 100 µl cell culture medium per well without supplements and 6 µl GenJet in 100 µl cell culture medium per well without supplements. Cells were incubated with the transfection reagents for approximately 20 h at 37°C and 5% CO_2_. Subsequently, transfection reagents were removed from the cells and exchanged for cell culture medium with supplements. The cells were incubated at 37°C and 5% CO_2_ for 24 h before seeding for testing.

### Homogeneous Time Resolved Fluorescence-Based cAMP Accumulation Assay

Twenty-four hours prior to performing the assay, cells were seeded into 384-well plates (Cellstar^®^ 384 well black µClear, Greiner Bio-One) with 8000 cells per well in 30 µl cell culture medium with supplements. The assay was performed using HTRF Gs protein dynamic kit reagents (Cisbio). After aspiration of the old medium, the cells were washed with 1x Tyrode buffer supplemented with 2 mM calcium chloride, followed by stimulation for 1 h (37°C and 5% CO_2_) with relaxin or forskolin in Tyrode buffer supplemented with 2 mM CaCl_2_ and 0.05% BSA, before cAMP-d2 (10 µl) and anti-cAMP-Cryptate (10 µl) were added to the solution. HTRF ratio (620 nm /665 nm) was measured after 1 h incubation at RT using a BMG PheraStar Plus and plotted against the log ligand concentration.

## Results

### Structural Insights From RXFP1 Homology Models

**FIGURE 2:**
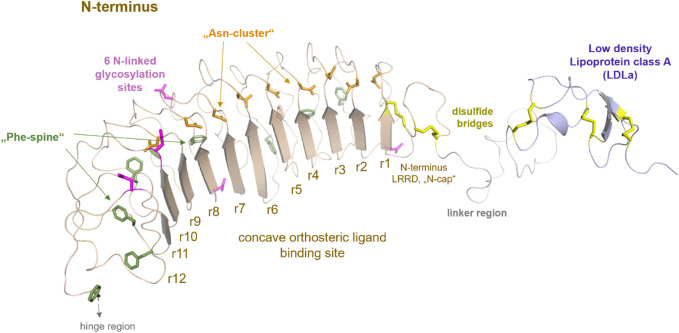
Putative structural details of extracellular RXFP1 modules. The extracellular RXFP1 N-terminus consists of four modules according to structural templates of homologous receptors and sequence properties: (i) The LDLa domain involved in signaling regulation (also complexed with a calcium ion (Hopkins et al., 2007)). (ii) The linker region between the LDLa and the LRRD. (iii) The LRRD with 12 repeating elements is each constituted by approximately 20−25 amino acids. The LRRD fold is stabilized by: 1. Two N-terminal cysteine bridges (N-cap); 2. A “Phe-spine” constituted by phenylalanines in the backbone of almost every repeat; 3. By an asparagine-enriched cluster (named Asn-cluster”) after the β-strand of each repeat, finally forming the concave β-sheet ligand binding site (Büllesbach and Schwabe, 2005). In the extracellular part, six potential glycosylation sites (Yan et al., 2008) are allocated (magenta sticks). (iv) The hinge region is short (∼30 amino acids) compared to other LGRs, such as glycoprotein hormone receptors (GPHR (Kleinau and Krause, 2009)), and contains one disulfide bridge.

So far known and postulated from the receptor sequence, RXFP1 consists of a TMD typical of GPCRs, comprising seven helices and six helix-interconnecting loops (Figure 1). However, the large extracellular part (Supplementary Figure S1, Figure 2) is atypical for class A GPCRs but is observed similarly in all evolutionary related LGR subtypes (Hsu et al., 2000). The extracellular part comprises an LDLa domain, a linker region between the LDLa and the LRRD, which is connected via a hinge region to TM1. The peptidic ligand relaxin is likely bound at two binding sites, the LRRD and the LDLa linker. This receptor part supposedly directly interacts with the ELs (Kong et al., 2013; Diepenhorst et al., 2014; Sethi et al., 2016b) in an as yet undeciphered mode of interaction. Yet, the N-terminal LDLa domain is mandatory for receptor signal regulation (Scott et al., 2006; Hopkins et al., 2007). The LDLa fold is stabilized by three cysteine bridges and an additional calcium ion binding site.

The linker region between the LDLa and the LRRD is somewhat unfolded, though the length as a spacer is important for correct receptor function (Nordström et al., 2009). Although this linker has no specific folding in the ligand-unbound state, certain residues are assumed to contribute to ligand binding and interaction of the ECD with the extracellular loops (Sethi et al., 2016b).

Two cysteine bridges stabilize the N-terminal fold of the LRRD, as known from the FSHR or TSHR (Kleinau et al., 2013). Of note, the LRRD shows specific structural properties involved in stabilizing the domain fold (Figure 2) (Kajava, 1998; Kobe and Kajava, 2001; Enkhbayar et al., 2004; Bella et al., 2008).

A structural fold for the short hinge region connecting the LRRD to the TMD cannot yet be predicted. Nonetheless, several LGRs share one intrinsic activation mechanism, described as the release of a “tethered ligand” (Kleinau et al., 2013; Schöneberg et al., 2016; Schulze et al., 2020). For GPHRs, several studies have demonstrated that parts of the extracellular region act as an intramolecular “tethered” ligand that is activated by hormone binding (Zhang et al., 2000; Vlaeminck-Guillem et al., 2002; Kleinau et al., 2004; Kleinau et al., 2013; Brüser et al., 2016). Such an intrinsic mechanism is also proposed for the class C LGRs, whereby the tethered ligand is located in the LDLa domain (Sudo et al., 2003; Scott et al., 2006; Hopkins et al., 2007) and is likely silent in the ligand-unoccupied state. Therefore, the correct (functional) spatial distance of the LDLa domain in RXFP1 to the potentially interacting ELs (Hopkins et al., 2007; Diepenhorst et al., 2014) may be strikingly dependent on the length of the LDLa-LRRD linker region (Sethi et al., 2016b). It is noteworthy that for class A and C LGRs, involvement of the EL2 in the signal transduction process has been suggested (Ryu et al., 1998; Nishi et al., 2002; Sudo et al., 2003; Kleinau et al., 2007; Kleinau et al., 2008; Dupakuntla et al., 2012; Sethi et al., 2016b).

### RXFP1 Construct Design

**FIGURE 3:**
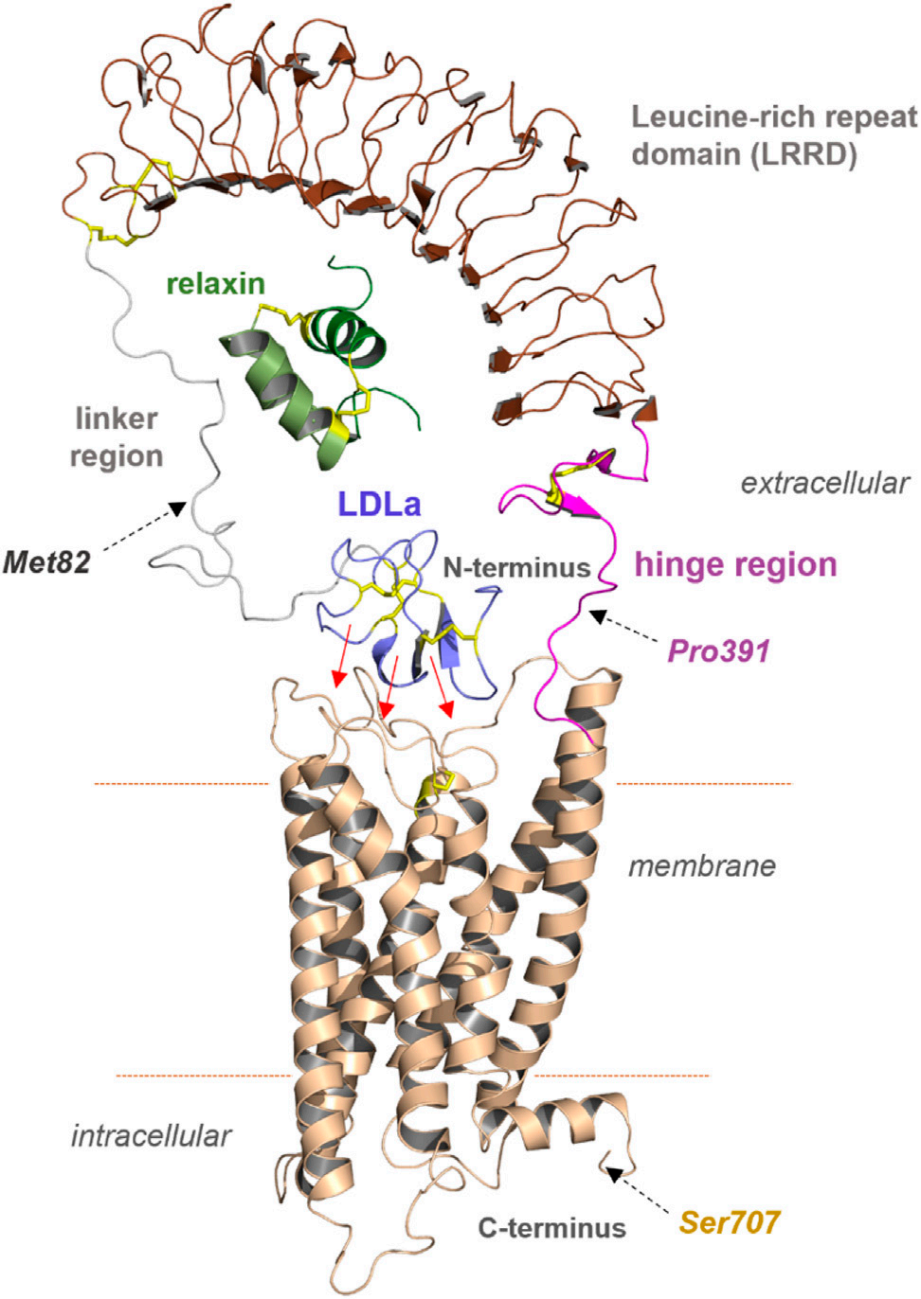
Predicted structural features of RXFP1. The assembled receptor parts and docked ligand relaxin visualizes principal features of the receptor and complex; however, it remains unknown how the diverse domains are arranged spatially to each other. Of note, the N-terminal LDLa domain is assumed to interact directly with extracellular loops or even parts of the TMD (indicated by red arrows). The endogenous ligand relaxin binds to both the LRRD and also potentially to the linker region simultaneously. Met82, Pro391, and Ser707 were used as breakpoints for the construction of diverse receptor variants.

**FIGURE 4:**
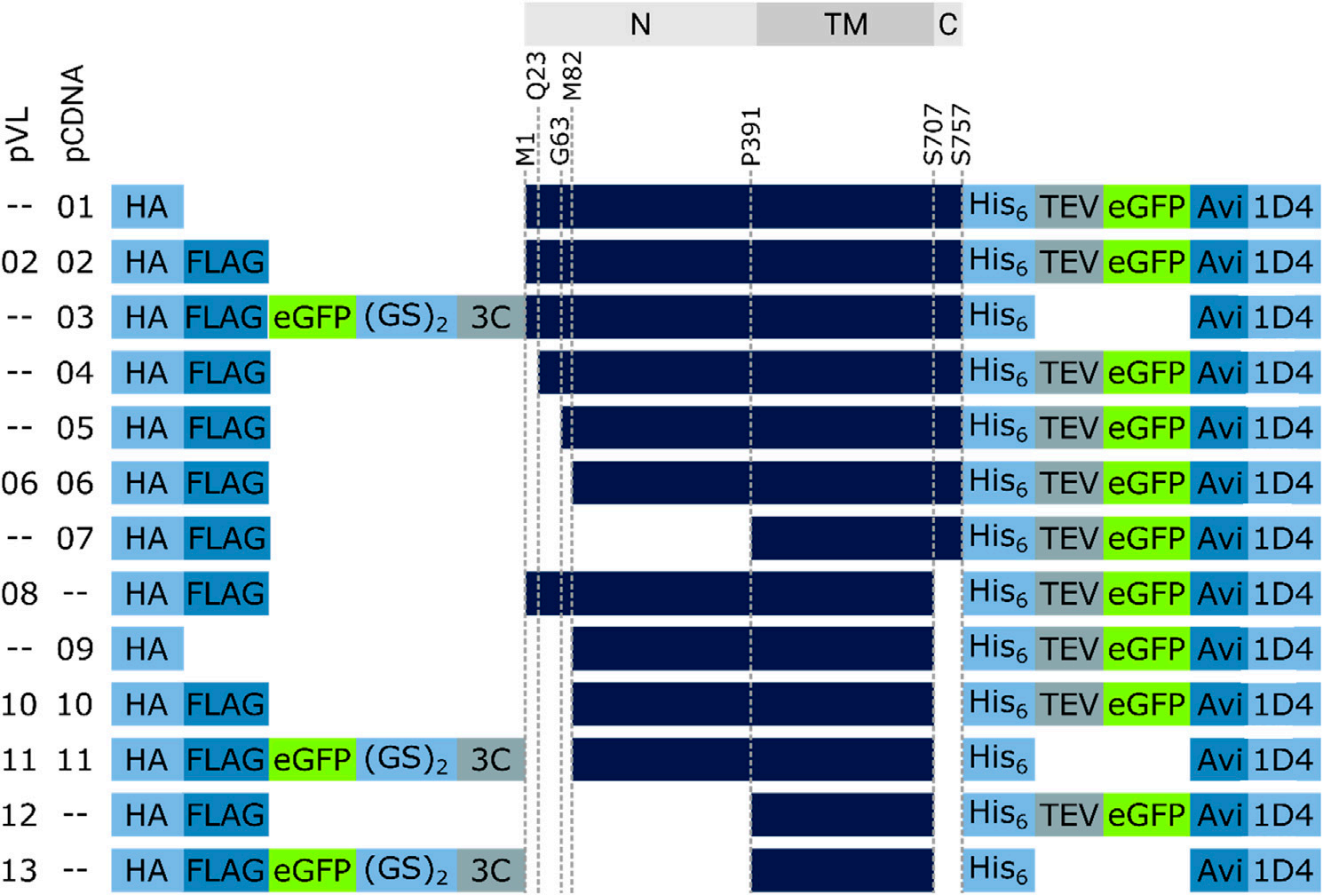
RXFP1 construct design. To assess the receptor region in which changes were made, the location is referenced above the constructs (gray bars, N = extracellular N-terminus, TM = transmembrane region, C = intracellular C-terminus). Several constructs do not differ in terms of GPCR sequence but in the position and number of tags used (HA = human influenza hemagglutinin signal sequence, GS = linker sequence, 3C & TEV = protease cleavage sequences, Avi = biotin-acceptor peptide, 1D4 = rhodopsin derived epitope tag).

One of our goals was to identify functionally active RXFP1 variants that can be isolated in sufficient quantity for further *in vitro* studies. For small-scale expression tests in *Spodoptera frugiperda* (*Sf*9) cells, the human RXFP1 sequence was cloned into the pVL1393 vector. Based on the structural-functional receptor model described above, N- and C-terminal receptor truncations were combined in several receptor variants. The constructs were designed under the following aspects: 1) deletion of the N-terminal LDLa domain (Met82 in the linker region), 2) deletion of the entire extracellular part (Pro391 as start residue), and 3) deletion of the receptor C-terminus (deletion at Ser707) (Figure 3 and Figure 4). For signaling experiments, human RXFP1 variants were cloned into the pCDNA6.2 expression vector.

### Screening of Different N- and C-Terminal Truncated RXFP1 Constructs

**FIGURE 5:**
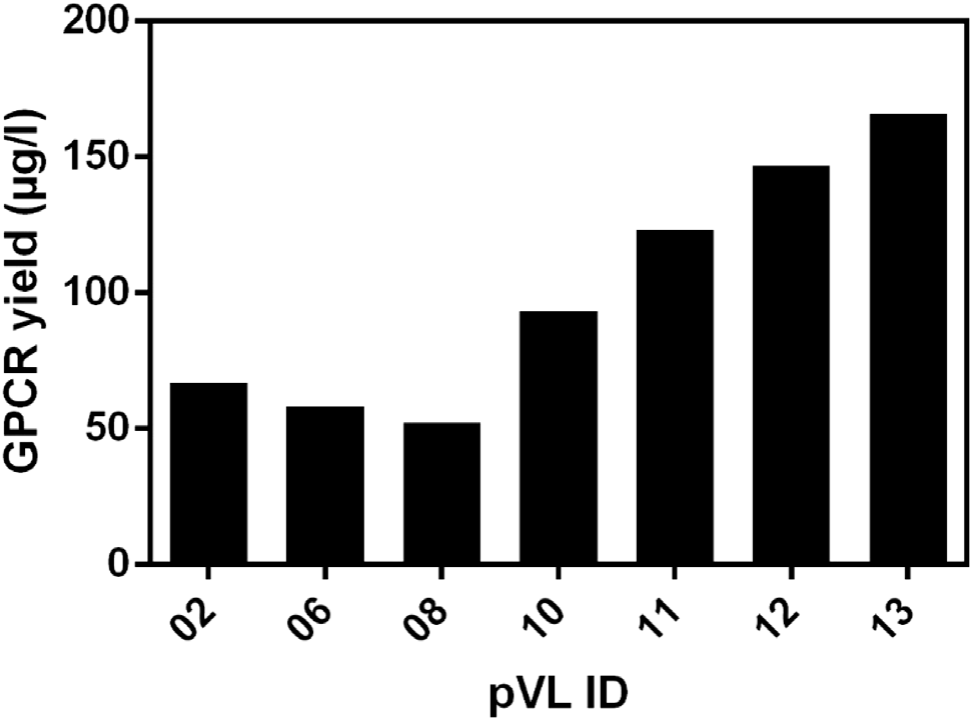
Comparison of the soluble receptor yield. Different RXFP1 variants (truncations and tag positions) were expressed in mid-scale and subsequently purified using the FLAG-tag for ANTI-FLAG^®^ M2 affinity chromatography. The final yield was determined by nanodrop microvolume UV-Vis spectrophotometer measurement and normalized to µg/L expression volume.

To estimate the amount of soluble receptor, 5 g of cell paste was purified using the FLAG-tag present in all constructs. To assess the impact of the changes on protein yield, all RXFP1variants were compared with the wild-type RXFP1 (WT- RXFP1) (pVL-02) construct (67 µg/L, Figure 5). The C-terminal truncation of residues 708 to 757 and the N-terminal truncation of amino acids 1 to 82 (and, therefore, the deletion of the LDLa domain) had no drastic impact on final protein yield (numbers pVL-06 & pVL-08). Only the combination of N- (with and without LRRD) and C-terminal deletions resulted in a receptor yield of over 100 µg/L (numbers pVL-10/11 & pVL-12/13). The direct comparison with respect to the eGFP position showed a significantly better yield when located at the N-terminus. In summary, both the shortening of the receptor on both ends and the N-terminal fusion to an eGFP tripled the final protein yield compared to the WT construct (numbers pVL-02 *vs.* pVL-13).

**FIGURE 6:**
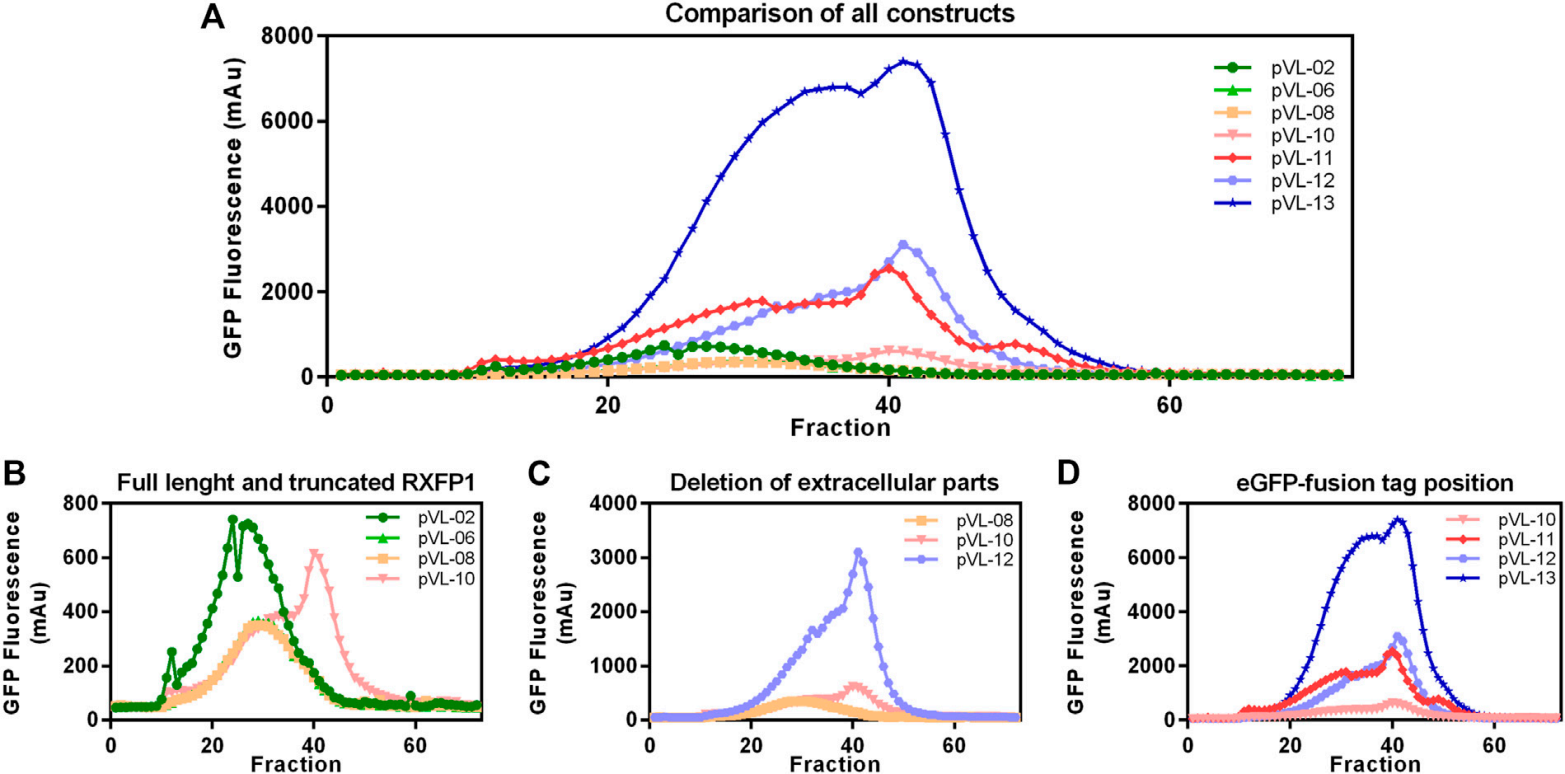
Construct characterization using F-SEC chromatography. Mid-scale ANTI-FLAG^®^ M2 affinity purified RXFP1 variants were analyzed based on their eGFP fluorescence signal. **(A)** Overview of all chromatograms. **(B)** Comparison between WT-RXFP1and N- and C-terminal truncations. **(C)** Impact of the deletion of extracellular parts. **(D)** Impact of the eGFP-fusion tag position.

Following the first purification step, clear effects of the different truncations on the yield of soluble RXFP1 were observed. Analytic F-SEC experiments of the elution fractions from the ANTI-FLAG^®^ M2 affinity purification revealed strong differences in the overall yield and the distribution between large soluble aggregates and monodisperse protein for different constructs (Figure 6A).

By comparing constructs with different N- and C- terminal truncations most constructs were shown to elute as soluble aggregates (pVL-02, pVL-06, pVL-08, Figure 6B). Only the combination of deleting the first 89 amino acids and the last 50 amino acids yielded monodisperse protein (pVL-10).

Since the removal of the LDLa domain had a positive effect on the monomeric GPCR RXFP1 yield, the LRRD was also removed in the next step (Figure 6C). Shortening the construct to the transmembrane region resulted in a significant increase in overall GFP fluorescence (600 mAu for pVL-10 *vs.* 3000 mAu for pVL-12) and a more prominent monomer protein peak. To investigate the effects of the eGFP position on the properties of the purified RXFP1 variant, the eGFP-fusion tag of constructs pVL-10 and pVL-12 was relocated to the N-terminus (pVL-11 and pVL-13). Direct comparison of the two truncated constructs shows an increase of the monomeric peak (fraction 40) when the eGFP-fusion tag was shifted from the C-terminus to the N-terminus (Figure 6D). Here, the best RXFP1 construct yield was obtained with the pVL-13 construct (FLA-eGFP-P391-S707-Avi) with a peak maximum of over 6000 mAu. If the focus had been placed only on the final protein yield, the construct pVL-13 with its high protein yield would have been a suitable candidate for all further experiments. However, since the ligand binding properties of RXFP1 should remain unaltered (e.g., for subsequent small-molecule screening), pVL-11 (FLA-eGFP-P391-S707-Avi) was used for further experimental work.

### Large-Scale Purification of RXFP1 Variant (Construct pVL-11, FLAG- eGFP-M82-S707-Avi)

**FIGURE 7:**
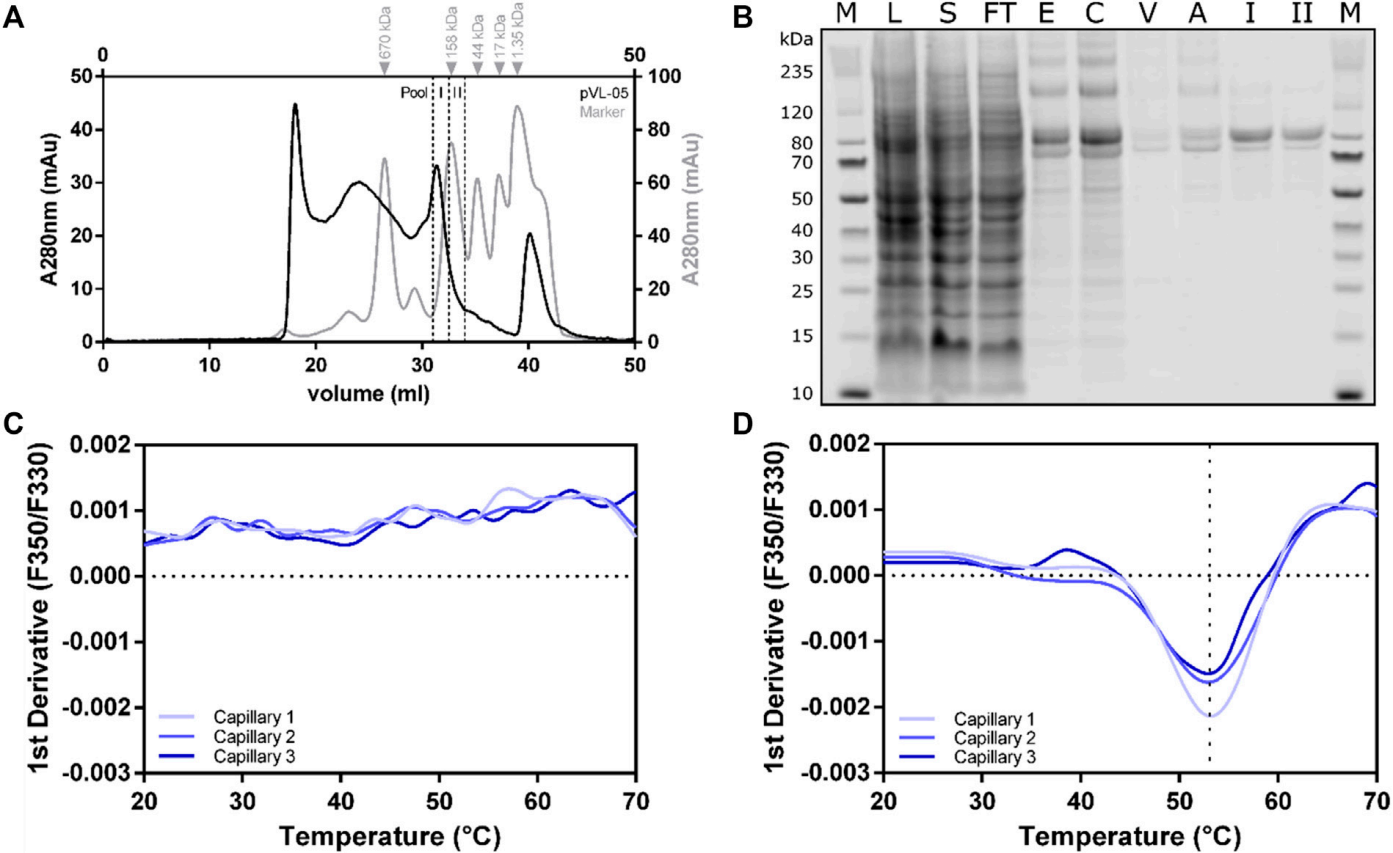
Large-scale purification of RXFP1 constructs. **(A)** SEC chromatogram of pVL-11 2L large-scale purification. **(B)** SDS-PAGE shows the course of pVL-11 purification, the expected size of the cut construct is 76 kDa (M = size standard, L = lysate, S = solubilizate, TF = flow through, E = M2 eluate, C= concentrated M2 eluate as SEC input, V = void volume peak, A = aggregate peak fraction, I = pool fraction I monomeric peak RXFP1, II = pool fraction II monomeric peak RXFP1). **(C)** nanoDSF melting curve of pVL-11 aggregate peak fraction. **(D)** nanoDSF melting curve of monomeric GPCR from the pool fraction I.

The cell pellet from a 2L *Sf9* expression culture was thawed in solubilization buffer (50 mM HEPES pH 7.5, 300 mM NaCl, 10% glycerol, 2 mM CaCl_2_, 0.5 mM EDTA, cOmplete™ protease inhibitor cocktail tablet (Roche Applied Science), 0.1 mM TCEP, and 2 mM biotin) without detergent. DDM/CHS was added to a final concentration of 2%, and cells were further disrupted by a douncing procedure. Solubilization was performed at 4°C for 1 h with gentle agitation. To separate the cell debris from the solubilized fraction, centrifugation was performed at 25 000 rcf for 45 min, and the supernatant was bound to 3 ml ANTI-FLAG^®^ M2 agarose resin for 2 h (at 4°C with overhead rotation). After separating the solubilizate from the ANTI-FLAG^®^ M2 agarose beads, with a rinsing step comprising 13 column volumes (CV) wash buffer (HEPES pH 7.5, 300 mM NaCl, 10% glycerol, 2 mM CaCl_2_, 0.1% DDM, 0.01% CHS, 0.1 mM TCEP) was performed. The receptor was subsequently eluted (50 mM HEPES pH 7.5, 150 mM NaCl, 10% glycerol, 0.1% DDM, 0.01% CHS, 0.1 mM TCEP, 200 µg/ml Flag peptide, and 5 mM EDTA) and separated into multiple fractions, with total volume of 6 ml. The eGFP fluorescence of all fractions was measured and combined accordingly. Following concentration using 100 kDa cut-off concentrator, the sample was finally polished using a Superose 6 10/30 increase column. Size-exclusion chromatogram revealed two main peaks in the region of interest between 20 and 35 ml (Figure 7A). Both peaks contained RXFP1 (as shown in the SDS-PAGE Figure 7B); however, only the second peak includes the monomeric receptor. The protein behaves smaller in the SDS-PAGE than the actual size. MS analytics (in gel digest) proved the integrity of the protein.

Thermal stability of the different F-SEC peak fraction pools (I and II) were analyzed with nano-differential scanning fluorimetry (nanoDSF) (Figures 7C,D). While the sample from the aggregated peak fraction had no melting curve, a melting temperature of approximately 53°C could be determined for the monomeric fraction I peak, which indicates a proper folded protein. Based on these results, the monomeric peak fraction I was used for subsequent ligand binding experiments using SPR.

### Characterization of Purified RXFP1 Proteins with Surface Plasmon Resonance (SPR)

**FIGURE 8:**
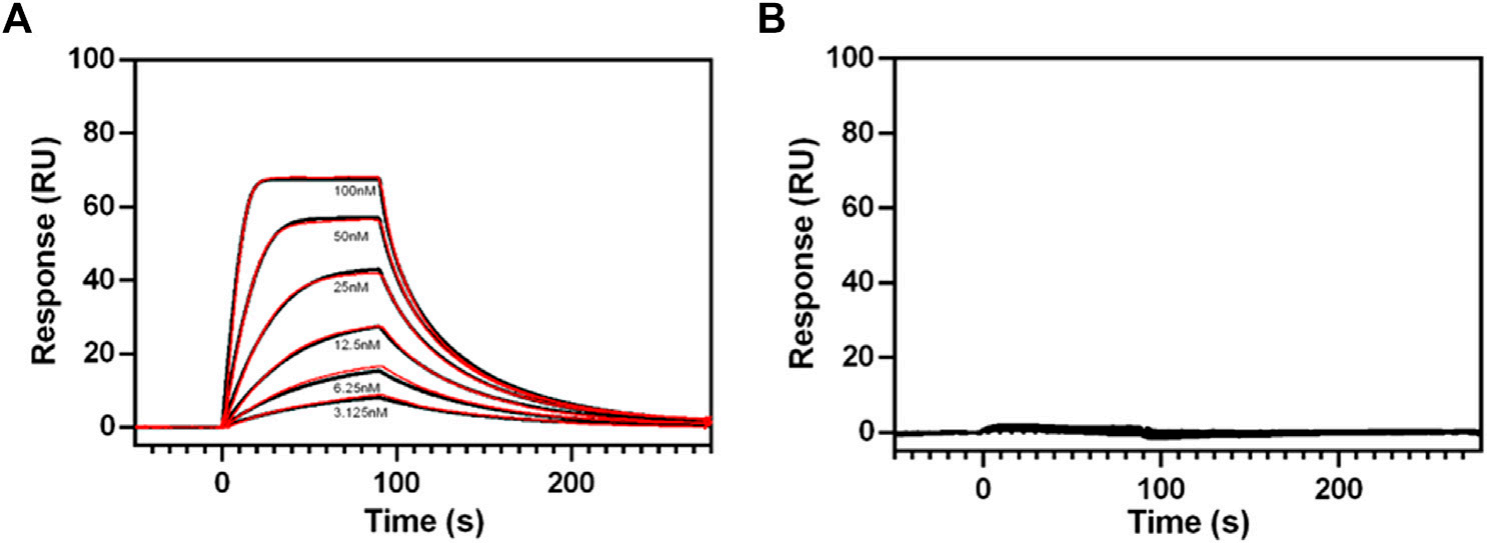
SPR sensograms of relaxin binding to different RXFP1 constructs. **(A)** Dose-dependent binding curves of relaxin (by adding with 3.125, 6.25, 12.5, 25, 50, and 100 nM relaxin concentration to the receptor fixed on the SPR chip) for RXFP1 pVL-11(FLAG-eGFP-M82-S707- AVI), and **(B)** for RXFP1 pVL-13(FLAG-eGFP-P391-S707-AVI). The red lines represent kinetic fits. Experiments were performed in triplicates.

Purified RXFP1 constructs pVL-11(FLAG-eGFP-M82-S707-Avi) and pVL-13 (FLAG-eGFP-P391-S7079-Avi) (purified using the same procedure) were used for SPR experiments. As both constructs carry a biotinylated Avi-tag at the C-terminus, they could be captured via the biotin on streptavidin sensor chip. The interaction with ligand relaxin was measured to prove that the purified protein is pharmacologically active, and the truncations did not lead to loss of relaxin binding capability. These SPR experiments clearly showed a concentration-dependent binding of relaxin to the purified pVL-11 construct with nanomolar affinity (*Kd* = 20 nM ±1,5) (Figure 8A), which is in accordance with previous reports (Chan et al., 2012; Halls et al., 2005). The RXFP1 construct without extracellular domain shows no dose-dependent ligand-response, confirming the importance of this domain for relaxin binding (Figure 8B), which is also in agreement with previous studies (Wu et al., 2016).

### cAMP Measurements Highlight the Importance of the LDLa Domain for RXFP1 Function

**FIGURE 9:**
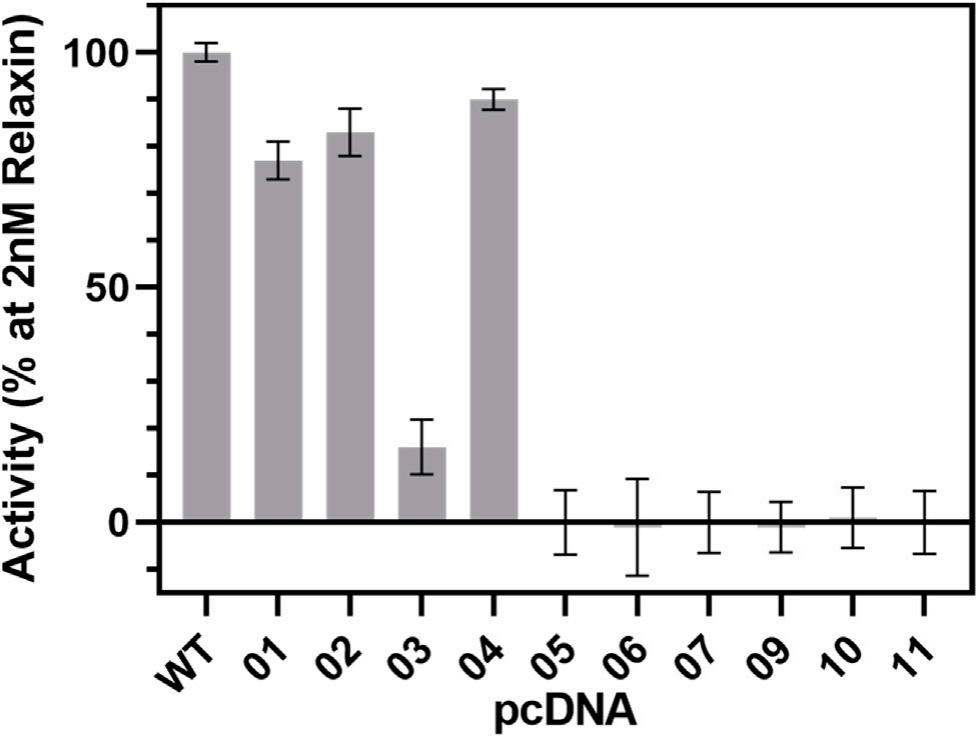
cAMP signaling of RXFP1 variants. All RXFP1 pCDNA constructs used for cAMP signaling assays are numbered according to Figure 4. Activity of all RXFP1 variants (WT and constructs 1−11) normalized to the cAMP signal of fully activated WT-RXFP1 using 2 nM relaxin and performed in quadruplicates.

To investigate the suitability of the different constructs in cellular assays, the cAMP signal was measured after stimulation with 2 nM relaxin (Figure 9). The activity of WT-RXFP1 (amino acids 1−757) without any tags was used as a reference for all other receptor variants.

N-terminal HA- or FLAG-tags combined with C-terminal His-eGFP-Avi-1D4 tags changed the functional activity of the construct slightly compared to WT-RXFP1, as shown by their ability to increase intracellular cAMP levels using 2 nM relaxin as an RXFP1 agonist. Removal of the first 23 amino acids did not dramatically affect cAMP accumulation. However, when the eGFP-fusion tag was shifted from the C-terminus to the N-terminus, the cAMP signal decreased to approximately 20% compared with the WT-RXFP1. Further shortening the N-terminus beyond the first 23 amino acids (with a resulting removal of the LDLa and LRR domains) resulted in a complete loss of signaling.

## Discussion

We here describe the design of various RXFP1 constructs with the aim of generating receptor variants suitable for functional and biophysical studies or structure determination. Several RXFP1 variants with different lengths and (technically required) tags were cloned and screened for sufficient protein expression. Most promising candidates were expressed in large-scale approaches accompanied by ligand binding studies and signaling assays. The expression of GPCRs is generally a difficult task, especially if they exert large extracellular domains in addition to the transmembrane helices. Indeed, the typical approach of shortening the N- and C-terminal regions of a GPCR resulted in increased expression in the case of RXFP1 as well as with enrichment in monomeric receptor fractions. Moving the eGFP-fusion tag to the N-terminus finally led to a sufficient yield for SPR analysis.

Moreover, we found that removing the LDLa domain leads to enhanced monomeric receptor expression without losing the ability to bind its natural ligand relaxin, even if the affinity decreases compared to the WT-RXFP1, which is consistent with other studies (Sethi et al., 2016b). However, this construct cannot be used for signaling experiments because cAMP accumulation no longer occurs after relaxin addition. Further truncation toward the transmembrane region of the receptor resulted in the highest possible receptor expression; however, neither relaxin binding nor a cAMP response to ligand stimulation was observable (Table 1).

**TABLE 1:** Summary of the results of this study.

RXFP1 constructs #	Yield monomeric RXFP1	Ligand Binding (SPR)	Signaling (cAMP)
pVL-1	n.d.	n.d.	+++
pVL-2	0	n.d.	+++
pVL-3	n.d.	n.d.	+
pVL-4	n.d.	n.d.	+++
pVL-5	n.d.	n.d.	0
pVL-6	0	n.d.	0
pVL-7	n.d.	n.d.	0
pVL-8	0	n.d.	n.d.
pVL-9	n.d.	n.d.	0
pVL-10	++	n.d.	0
pVL-11	+++	+++	0
pVL-12	++++	n.d.	n.d.
pVL-13	+++++	0	n.d.

Based on small- and mid-scale expressions, the monomeric receptor yield was scored between low (+) and very high (+++++). The SPR measurements showed either a binding of relaxin in the nanomolar range (+++) or could not measure binding (0). Signaling measurements based on cAMP showed either no (0), moderate (+), or responses comparable to the WT-RXFP1 (+++) after addition of 2 nM relaxin. Measurements that were not performed are marked with n.d.

RXFP1 variants pVL-11 (FLAG-eGFP-M82-S707-Avi) and pVL-13 (FLAG-eGFP-P391-S7079-Avi) are particularly suitable for SPR ligand screening and subsequent structural elucidation. In the case of pVL-11, ligands could be potentially found that, like relaxin, require the LRRD for high-affinity binding. In the case of construct pVL-13, it should be possible to identify compounds that bind in the transmembrane region. For the cAMP assay, the construct would then have to be extended by the LDLa module.

Despite these described efforts in our study, some limitations and future needs should be addressed. First, RXFP1 is known to be a receptor that can activate several signaling pathways besides cAMP (Gs), such as ERK1/2, p38MAPK, Gi3, or cGMP (Halls et al., 2007; Bathgate et al., 2013; Kocan et al., 2017). These signaling pathways are of utmost importance to capture the full pharmacological and physiological spectrum of this receptor. Therefore, in addition to the cAMP accumulation studied here, it will be interesting to test signaling-competent receptor variants for their ability to activate the extended set of signaling pathways. This is also particularly interesting because the cell systems used here (*Sf*9 insect cells for expression, HEK293 cells for functional characterization) differ from cell types of organs (such as ovary, testis, cervix, breast, endometrial epithelial cells) that naturally express this receptor (Kong et al., 2010) in terms of the amount of G-proteins or other intracellular factors and effectors. In addition, there are different glycosylation patterns for proteins in insect cells (e.g. (Marada et al., 2015), which is also relevant for RXFP1 with six putative glycosylation sites. These sites have effects on receptor expression and cAMP accumulation, but not on ligand binding (Yan et al., 2008). This, in turn, suggests that experimental approaches with “artificial” cell systems overexpressing the receptor may vary in terms of the nature of glycosylation or intracellular response to stimulation. These circumstances must generally be taken into account for *in vitro* data obtained, which also applies to our study. However, the cell systems used here are currently common experimental tools (e.g. (Shukla et al., 2006)) for the subsequent structural and biophysical methods, which at least allow comparability between different experimental studies. Finally, it should be mentioned that RXFP1 is capable of binding three different relaxin proteins (Patil et al., 2017) and that our relaxin H2-binding competent constructs designed here should be tested for the binding of other ligand-peptides to assess the binding specificities in detail.

**FIGURE 10:**
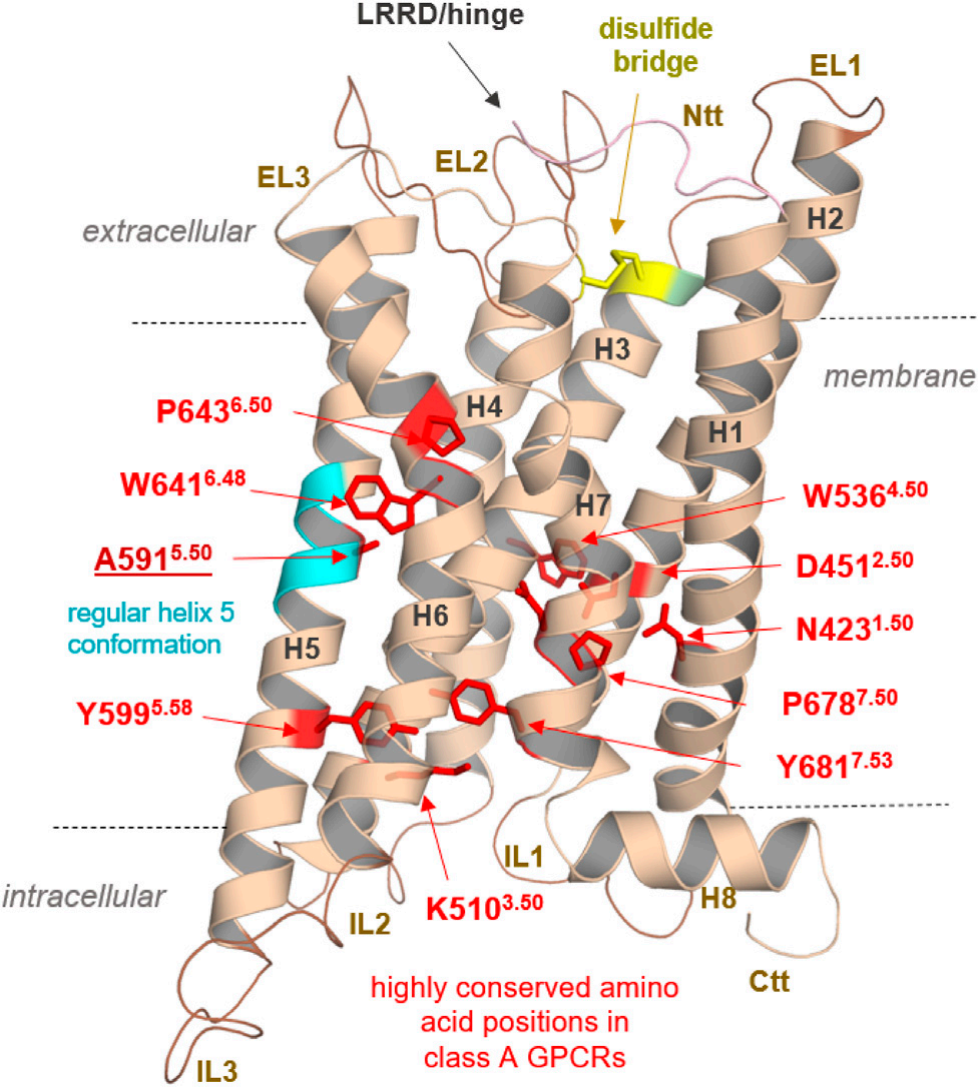
Specificities of RXFP1 and common class A GPCR properties. Typical highly conserved class A GPCR amino acids are highlighted as red sticks in the TMD model of the RXFP1. They are responsible for specific receptor functions as structural fold, expression, and signal transduction. In addition, they determine structural features in the helices (as the prolines, e.g., P643^6.50^), or they participate as intramolecular switches (as W641^6.48^) regulating the transition between active and inactive state conformations (D451^2.50^). Of note, the TMH5 conformation is likely regular compared to most other class A GPCRs with a proline-induced kink (cyan helix region) because RXFP1 has an alanine at this position. Superscripted numbers according to the unifying Ballesteros & Weinstein numbering for class A GPCRs (Ballesteros and Weinstein, 1995)*.* An additional structural factor of importance shared between the LGRs is homo- or hetero-oligomerization, a widely recognized property of many GPCRs (e.g., (Smith and Milligan, 2010; Tena-Campos et al., 2014)), including receptors of relevance in endocrinology (Kleinau et al., 2016). Cooperative effects between receptor monomers arranged as dimers or oligomers (homomers) are long known to be obligatory and impact LGR groups A and C ligand binding properties (Chazenbalk et al., 1996; Urizar et al., 2005; Svendsen et al., 2009; Zoenen et al., 2012). Furthermore, interactions with non-LGR receptors (heteromerization), such as angiotensin receptors (AT_1_R and AT_2_R), have been identified *in vivo*, specifically for RXFP1 (Chow et al., 2014; Chow et al., 2019). This fact drastically widens the spectrum of the potential functional and physiological importance of this receptor. Finally, the RXFP1 is a highly interesting and pharmacologically relevant class A GPCR with unique structural and functional features, which needs further advanced experimental elucidation.

Anyhow, our findings could pave the way for further structural elucidation by protein X-ray crystallography or cryo-electron microscopy methods of this unique GPCR, which shows diverse, interesting structural features merely by analyses of the sequence (Supplementary Figure S1) and obtained homology models. These specificities are related to the module-like architecture of the RXFP1, the interplay between LDLa and TMD, or specific amino acids in the transmembrane domain (Figure 10).

## Data Availability

The original contributions presented in the study are included in the article/Supplementary Material, further inquiries can be directed to the corresponding authors.
