# Supplementary material for: Expression and Characterization of Relaxin Family Peptide Receptor 1 Variants
Source: Front Pharmacol. 2022 Jan 28;12:826112. doi: 10.3389/fphar.2021.826112 (PMC8832513; doi:10.3389/fphar.2021.826112)
Supplement: Supplementary file 1 [file DataSheet1.docx]

Supplementary Material

Expression and Characterization of Relaxin Family Peptide Receptor 1 Variants

**David Speck ^1^, Gunnar Kleinau ^1^, Mark Meininghaus ^2^, Antje Erbe ^3, 4^, Alexandra Einfeldt ^3, 4^, Michal Szczepek ^1^, Patrick Scheerer ^1, 5 *^, and Vera Pütter ^3, 4 *^**

^1^ Charité – Universitätsmedizin Berlin, corporate member of Freie Universität Berlin and Humboldt-Universität zu Berlin, Institute of Medical Physics and Biophysics, Group Protein X-ray Crystallography and Signal Transduction, Berlin, Germany

^2^ Bayer AG, Research & Development, Pharmaceuticals, Wuppertal, Germany

^3^ Bayer AG, Research & Development, Pharmaceuticals, Berlin, Germany

^4^ NUVISAN ICB GmbH, Berlin, Germany

^5^ DZHK (German Centre for Cardiovascular Research), partner site Berlin. Berlin, Germany

*** Correspondence:**Patrick Scheerer: patrick.scheerer@charite.de

and

Vera Pütter: vera.puetter@nuvisan.com

**Supplemental Material and Methods**

***Homology modeling procedures (continued)***

Due to the higher sequence similarities in this protein part, we hypothesized that the RXFP1 and opsin share a more similar conformation and localization of the EL2 relative to the other ELs and to the 7TMD as observed in the β2 adrenoreceptor. Therefore, the β2-adrenoreceptor EL2 was substituted with the already determined opsin EL2 structure under superimposition of both templates.

Of note, the kinked β2-adrenoreceptor TMH5 was manually replaced by a regular α-helix. In fact, a highly conserved proline in this helix at position 5.50 of class A GPCRs (unifying Ballesteros & Weinstein numbering [1]) is naturally substituted by an alanine in RXFP1, as also observed for other members of the LGR sub-class ([2; 3]). A proline in TMH5 supports a helical kink and bulge and distorts the regular helix geometry by steric conflicts with the preceding residue and the loss of a backbone hydrogen bond [4]. In conclusion of the missing proline in the RXFP1 TMH5 we assume that - in contrast to the template structure – this conformation is rather a regular helix.

*Amino acid substitutions and model composition* - The amino acid residues of the chimeric active and inactive state 7TMD receptor-conformation templates and the FSHR N-terminus were mutated with the corresponding RXFP1 amino acid sequence, respectively. These rough and partial homology models were generated with the software SYBYL-X 2.0 (Certara, NJ, US) and energetically minimized with the implemented Amber99 force field until converging at a termination gradient of 0.05 kcal/mol*Å.

The relaxin-3 (PDB ID: 2FHW, [5]) structure was manually complexed with the LRRD/hinge region model by using known interaction constraints between the receptor and the agonistic ligands as reported and suggested previously (e.g. [5; 6; 7; 8]), followed by an energetic minimization.

The N-terminal LDLa/linker region and the LRRD/hinge/relaxin-3 complex were fused at positions 94-95. This completed extracellular part was fused with the active 7TMD conformation model at position 421. Of note, the orientation and spatial distances between the extracellular receptor parts relative to the 7TMD cannot be predicted appropriately based on the fact, that detailed structural data received by protein X-ray crystallography or cryo-electron microscopy are not available so far. In conclusion, models showing a full length LGR conformation are just approximations especially with respect to the specific domain assembling.

Anyhow, our assembled complex model was optimized by energy minimization under constrained backbone atoms (the AMBER F99 force field was used), followed by a 2 ns molecular dynamics simulation (MD) with constraint backbone atoms of the transmembrane helices and the LRRD β-sheet.

Moreover, during this simulation the relaxin-3 was fixed to the LRRD by particular distance constraints between known interacting amino acid side chains. Finally, due to previous and recent studies on the RXFP1 activation mechanism [9; 10; 11; 12], which revealed that the LDLa domain might be located between the extracellular loops and may function as a signaling hub activated by endogenous ligand binding, we placed the LDLa domain close to the loops by using short distance constraints between the LDLa and the ELs during a 2ns MD simulation with just a released linker region. In this current study we did not attend to simulate detailed intramolecular interactions involved in receptor activity regulation (e.g. supposed LDLa - EL interactions), but rather were interested on the modular architecture of RXFP1 for correct protein variant design. The entire complex was then energetically minimized without any constraint until converging at a termination gradient of 0.05 kcal/mol*Å. The inactive state model of the RXFP1 based on the β2-adrenoreceptor [13] was minimized without any constraint.


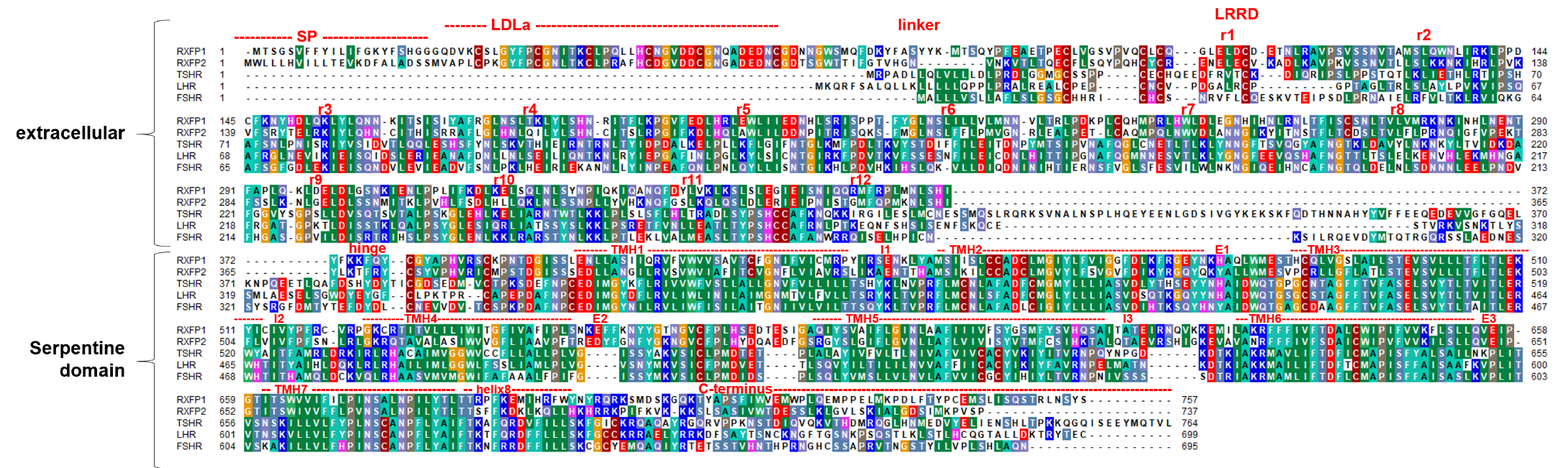


***Suppl. Fig. S1: Alignment comparison of different LGRs, including type C LGRs - LGR7 (*RXFP1) *and LGR8 (RXFP2) - and type A LGRs (TSHR, FSHR and LHR).*** *Annotated are structural properties predicted by sequence comparison with already determined structures as the FSHR extracellular domain. The alignment was visualized using the software BioEdit [14]. Background colors indicating conservation: black, proline; blue, positively charged; cyan/green, aromatic and hydrophobic; green, hydrophobic; red, negatively charged; gray, hydrophilic; dark red, cysteines; and magenta, histidine. SP - signal peptide, r - repeat, E - extracellular loop, I - intracellular loop, TMH – transmembrane helix, LDLa – Low density Lipoprotein class A (LDLa)*

**References Supplemental Material**

[1] J.A. Ballesteros, H. Weinstein, and C.S. Stuart, Integrated methods for the construction of three-dimensional models and computational probing of structure-function relations in G protein-coupled receptors, Methods in Neurosciences, Academic Press, 1995, pp. 366-428.

[2] G. Kleinau, I. Hoyer, A. Kreuchwig, A.K. Haas, C. Rutz, J. Furkert, C.L. Worth, G. Krause, and R. Schulein, From molecular details of the interplay between transmembrane helices of the thyrotropin receptor to general aspects of signal transduction in family a G-protein-coupled receptors (GPCRs). J Biol Chem 286 (2011) 25859-71.

[3] G. Kleinau, S. Neumann, A. Gruters, H. Krude, and H. Biebermann, Novel insights on thyroid-stimulating hormone receptor signal transduction. Endocr Rev 34 (2013) 691-724.

[4] H. Reiersen, and A.R. Rees, The hunchback and its neighbours: proline as an environmental modulator. Trends Biochem Sci 26 (2001) 679-84.

[5] K.J. Rosengren, F. Lin, R.A. Bathgate, G.W. Tregear, N.L. Daly, J.D. Wade, and D.J. Craik, Solution structure and novel insights into the determinants of the receptor specificity of human relaxin-3. J Biol Chem 281 (2006) 5845-51.

[6] E.E.S. Büllesbach, Christian, The Relaxin Receptor-binding Site Geometry Suggests a Novel Gripping Mode of Interaction. The Journal of Biological Chemistry (2000).

[7] E.E.S. Büllesbach, Christian, The Trap-like Relaxin-binding Site of the Leucine-rich G-protein-coupled Receptor 7. The Journal of Biological Chemistry (2005).

[8] E.E.Y. Büllesbach, Su; Schwab, Christian The Receptor-binding Site of Human Relaxin II - A Dual Prong-Binding Mechanism. The Journal of Biological Chemistry (1992).

[9] N.A. Diepenhorst, E.J. Petrie, C.Z. Chen, A. Wang, M.A. Hossain, R.A. Bathgate, and P.R. Gooley, Investigation of interactions at the extracellular loops of the relaxin family peptide receptor 1 (RXFP1). J Biol Chem 289 (2014) 34938-52.

[10] R.C. Kong, E.J. Petrie, B. Mohanty, J. Ling, J.C. Lee, P.R. Gooley, and R.A. Bathgate, The relaxin receptor (RXFP1) utilizes hydrophobic moieties on a signaling surface of its N-terminal low density lipoprotein class A module to mediate receptor activation. J Biol Chem 288 (2013) 28138-51.

[11] D.J. Scott, S. Layfield, Y. Yan, S. Sudo, A.J. Hsueh, G.W. Tregear, and R.A. Bathgate, Characterization of novel splice variants of LGR7 and LGR8 reveals that receptor signaling is mediated by their unique low density lipoprotein class A modules. J Biol Chem 281 (2006) 34942-54.

[12] A. Sethi, S. Bruell, N. Patil, M.A. Hossain, D.J. Scott, E.J. Petrie, R.A.D. Bathgate, and P.R. Gooley, The complex binding mode of the peptide hormone H2 relaxin to its receptor RXFP1. Nat Commun 7 (2016) 11344.

[13] V. Cherezov, D.M. Rosenbaum, M.A. Hanson, S.G. Rasmussen, F.S. Thian, T.S. Kobilka, H.J. Choi, P. Kuhn, W.I. Weis, B.K. Kobilka, and R.C. Stevens, High-resolution crystal structure of an engineered human beta2-adrenergic G protein-coupled receptor. Science 318 (2007) 1258-65.

[14] T.A. Hall, BioEdit: a user-friendly biological sequence alignment editor and analysis program for Windows 95/98/NT. Nucleic Acids Symposium Series Series 41 (1999) 95-98.
